# Supplementary material for: An Implantable Magneto-Responsive Poly(aspartamide) Based Electrospun Scaffold for Hyperthermia Treatment
Source: Nanomaterials (Basel). 2022 Apr 26;12(9):1476. doi: 10.3390/nano12091476 (PMC9101327; doi:10.3390/nano12091476)
Supplement: Supplementary file 1 [file nanomaterials-12-01476-s001.zip › nanomaterials-1674759-supplementary.pdf]

Supplementary material of

An Implantable Magneto-Responsive Poly(Aspartamide) Based Electrospun Scaffold for Hyperthermia Treatment

Tamás Veres [1,†], Constantinos Voniatis [1,2,†], Kristóf Molnár [1], Dániel Nesztor [3], Daniella Fehér [4], Andrea Ferencz [4], Iván Gresits [5], György Thuróczy [6], Bence Gábor Márkus [7], Ferenc Simon [8], Norbert Marcell Nemes[9], Mar García-Hernández [9], Lilla Reiniger [10], Ildikó Horváth [5], Domokos Máthé [5,11], Krisztián Szigeti [5], Etelka Tombácz [3,12] and Angela Jedlovszky-Hajdu [1,*]

1 Laboratory of Nanochemistry, Department of Biophysics and Radiation Biology, Semmelweis University, Budapest, 1089, Hungary; veres454@gmail.com (T.V.); constantinosvoniatis@gmail.com (C.V.); molnar.182@osu.edu (K.M.)

2 Department of Surgery, Transplantation and Gastroenterology, Semmelweis University, Budapest, 1082, Hungary;

3 Department of Food Engineering, University of Szeged, Szeged, 6725,Hungary; nesztor@chem.u-szeged.hu (D.N.); e.tombacz@chem.u-szeged.hu (E.T.)

4 Heart and Vascular Centre, Department of Surgical Research and Techniques, Semmelweis University, Budapest, 1122, Hungary; daniella.feher@gmail.com (D.F); ferencz.andrea@med.semmelweis-univ.hu (A.F.)

5 Department of Biophysics and Radiation Biology, Semmelweis University, Budapest, 1094, Hungary; gresits.ivan@gmail.com (I.G.); horvath.ildiko@med.semmelweis-univ.hu (I.H.); mathe.domokos@med.semmelweis-univ.hu (D.M.); krisztian.szigeti@gmail.com (K.S.)

6 NRIRR "Frédéric Joliot-Curie" National Research Institute for Radiobiology and Radiohygiene1221 Budapest, Hungary; thuroczy@hp.osski.hu

7 Stavropoulos Center for Complex Quantum Matter, Department of Physics and Astronomy, University of Notre Dame, Notre Dame, IN 46556, USA; bmarkus@nd.edu

8 Institute of Physics, Budapest University of Technology and Economics, PoBox 91, 1521 Budapest,
Hungary, Wigner Research Centre for Physics Economics, Budapest, 1121, Hungary; simon.ferenc@ttk.bme.hu

9 GFMC, Departamento de Física de Materiales, Universidad Complutense de Madrid, 28040 Madrid, Spain; nmnemes@fis.ucm.es (N.M.N.); marmar@icmm.csic.es (M.G.-H.)

10 Department of Pathology
and Experimental Cancer Research, Semmelweis University, Budapest, 1085,Hungary; reiniger.lilla@med.semmelweis-univ.hu

11 Hungarian Center of Excellence for Molecular Medicine (HCEMM), In Vivo Imaging Advanced Core
Facility, Semmelweis University Site, 1094 Budapest, Hungary

12 Soós Ernő Water Technology Research and Development Center, University of Pannonia, Nagykanizsa, 8800, Hungary

* Correspondence: hajdu.angela@med.semmelweis-univ.hu

† These authors contributed equally to the project, therefore they both should be considered as first authors of this paper.

Preparation of the magnetic membranes– crosslinking and the synthesis of iron oxide particles

Optimization of the samples was done the following way. PSI membranes were cut in a 16 mm diameter cylinders, they were first treated with 0.5 M DAB/EtOH for 1 or 2-hour intervals to create the crosslinks between polymer chains inside the fibres. Next, samples were washed with ultrapure (UP) water, and soaked into Fe(II)-Fe(III)-chloride solution for 30 minutes, 3 hours or 24 hours. After washing the samples with UP water again, they were treated with 3 M NaOH solution for 10 or 30 minutes (Table S1).

The nomenclature of the samples is the following: PSI-DAB-Magn-1st time point (corresponds to the DAB/EtOH treatment)-2nd time point (corresponds to the Fe-chloride treatment)-3rd time point (corresponds to the NaOH treatment). We picked the PSI-Magn-1h-30min-30min as a standard sample based on the results in the main article.

		**Fe(II)-Fe(III)-chloride treatment**				
		**30min**	**3h**	**24h**		
**DAB/EtOH treatment**	**1h**	1h30min10min			**10min**	**NaOH treatment**
		1h30min30min			**30min**	
	**2h**	2h30min10min	2h3h10min	2h24h10min	**10min**	

Table S1: Experimental setups of the samples: the time intervals refer to DAB/EtOH, Fe(II)-Fe(III)-chloride and NaOH treatment times respectively.

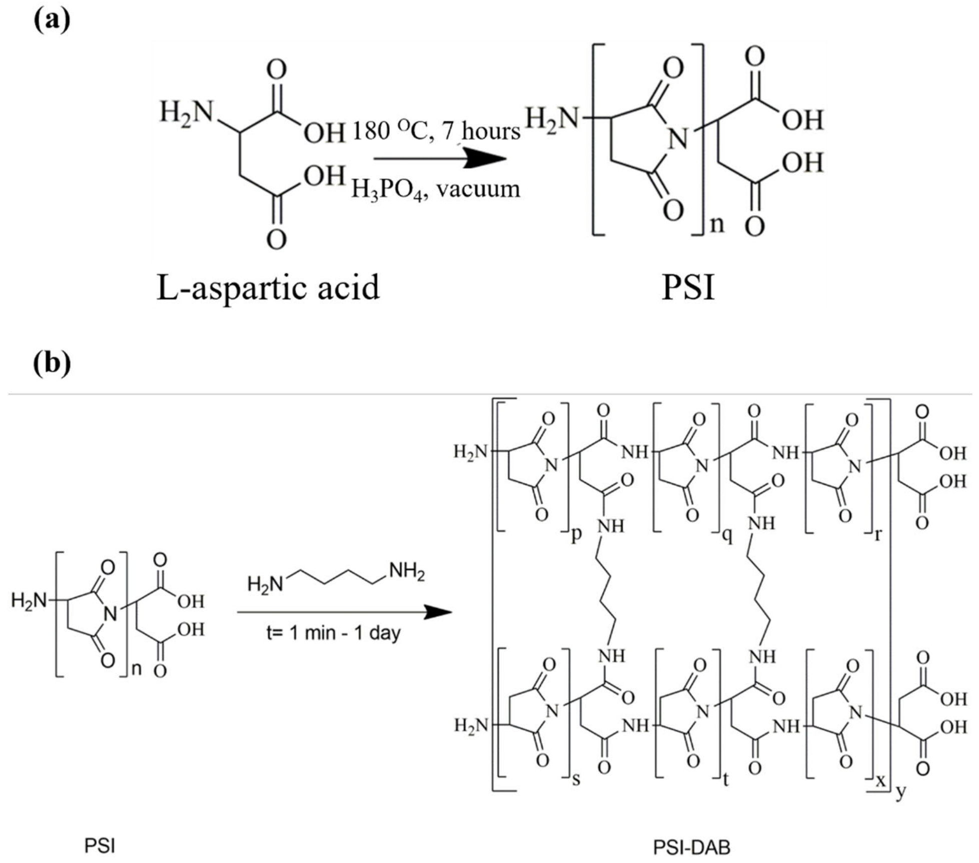

Figure S1: (**a**) PSI is made by the thermal polycondensation reaction of L-aspartic acid. (**b**) DAB creates crosslinks between PSI chains by opening the imide ring

(a)

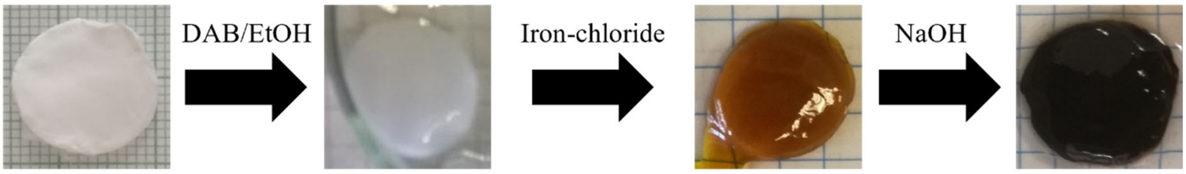

(b)

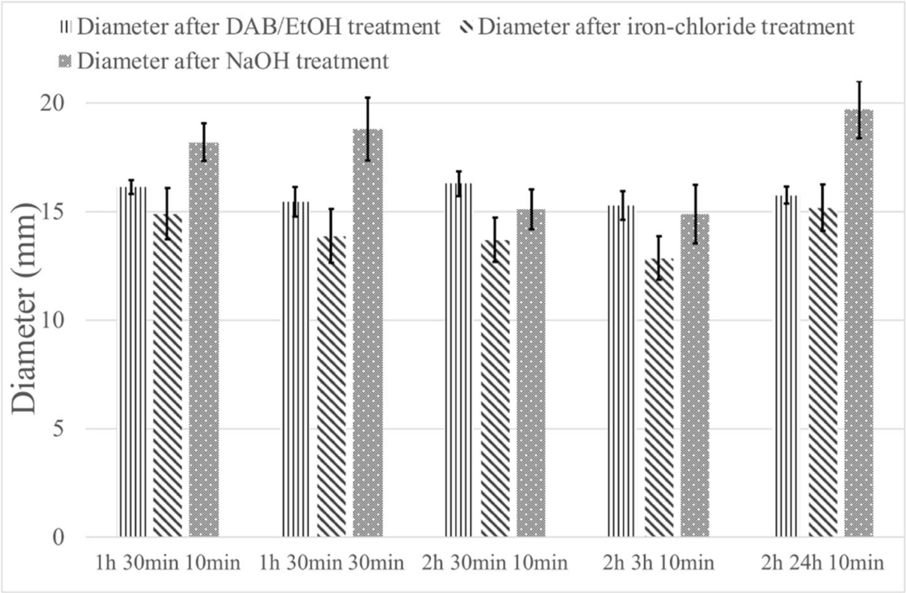

(c)

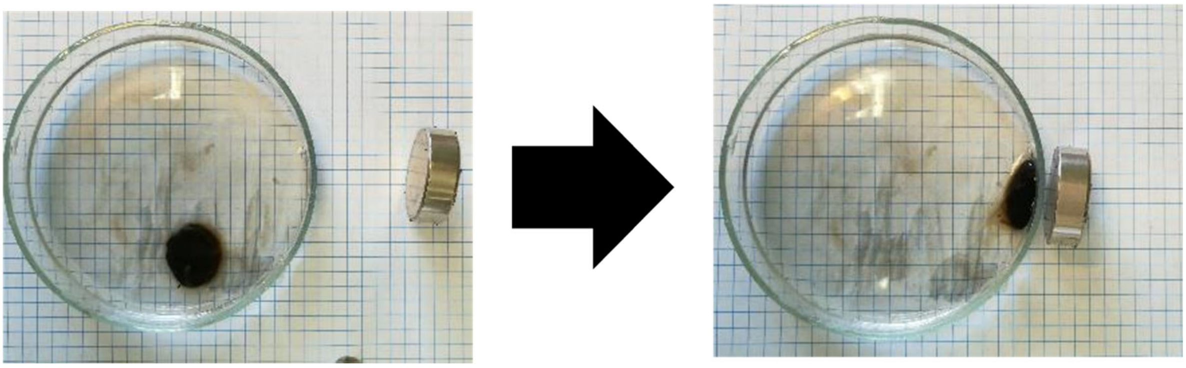

Figure S2: (**a**) Photos of samples after each treatment step. (**b**) Change in diameter of the samples after each treatment step. (**c**) Samples react to external static magnetic field.

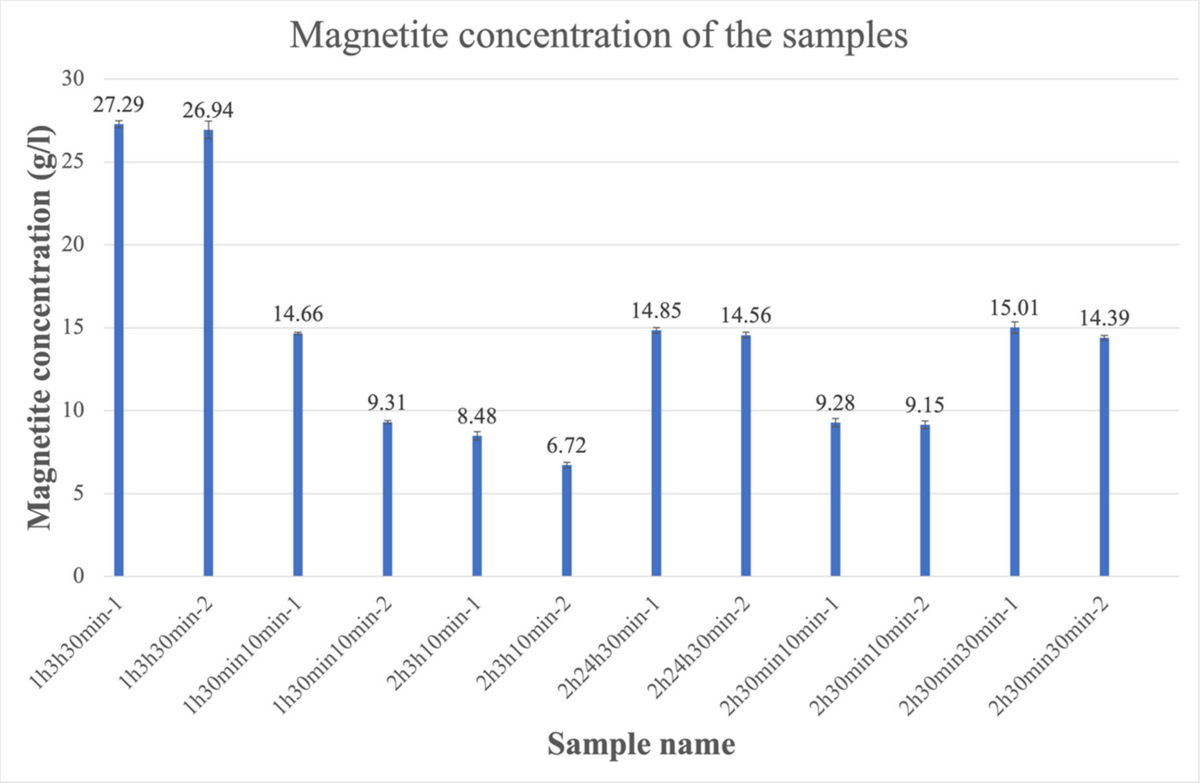

Figure S3 Iron oxide content of the meshes.

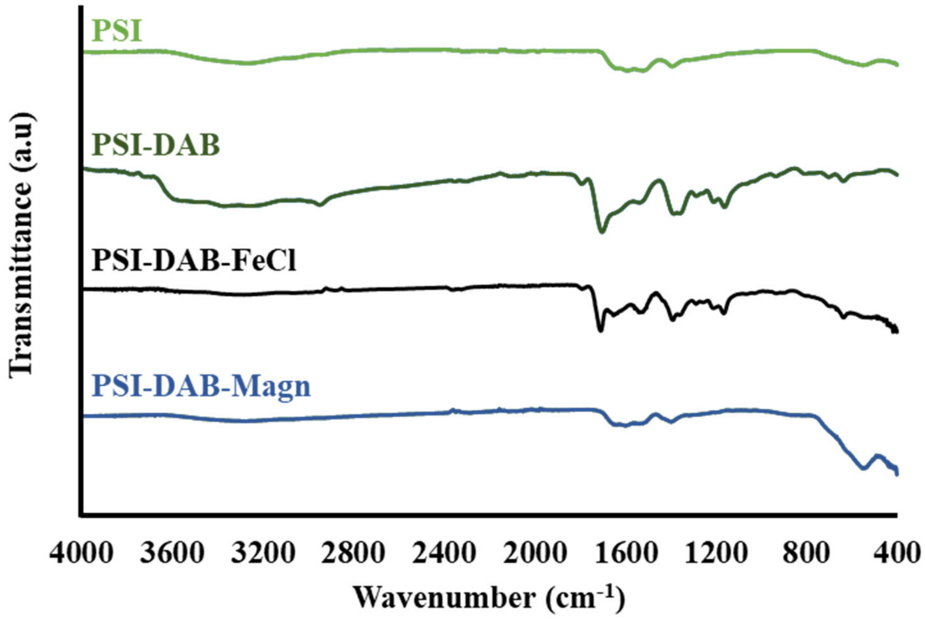

Figure S4: FTIR spectra of the PSI-DAB-Magn sample taken after each treatment step.

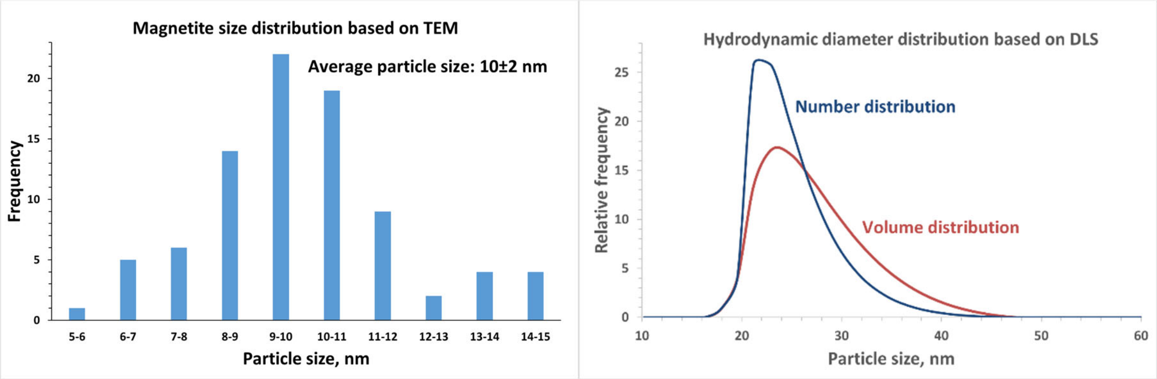

Figure S5: The size distribution of the iron oxide nanoparticles based on TEM and DLS measurements. The PDI was 0.2 based on the DLS.

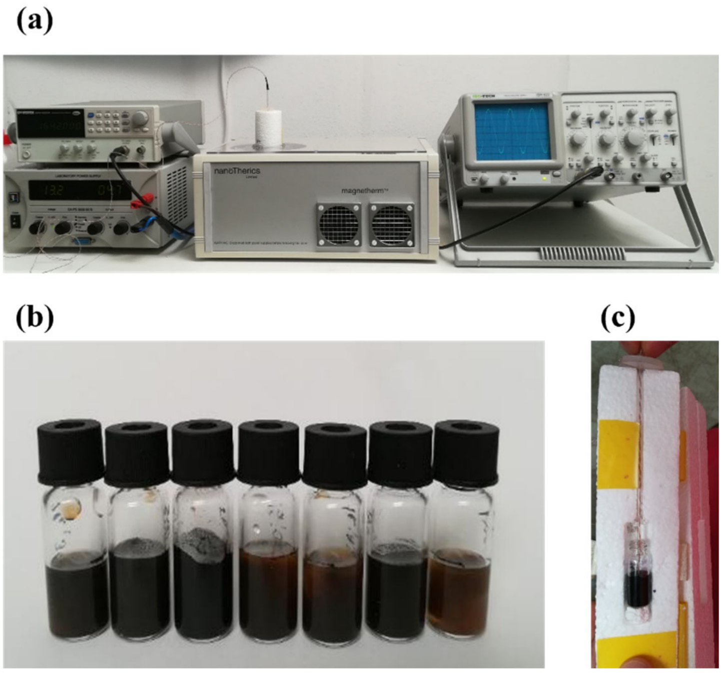

Figure S6: (**a**) Picture of MagneTherm 1.5 magnetic hyperthermic instrument (**b**) Samples in the 2ml vials for hyperthermic measurements (**c**) The sample with the thermometer during the hyperthermic measurement in the isolation chamber.

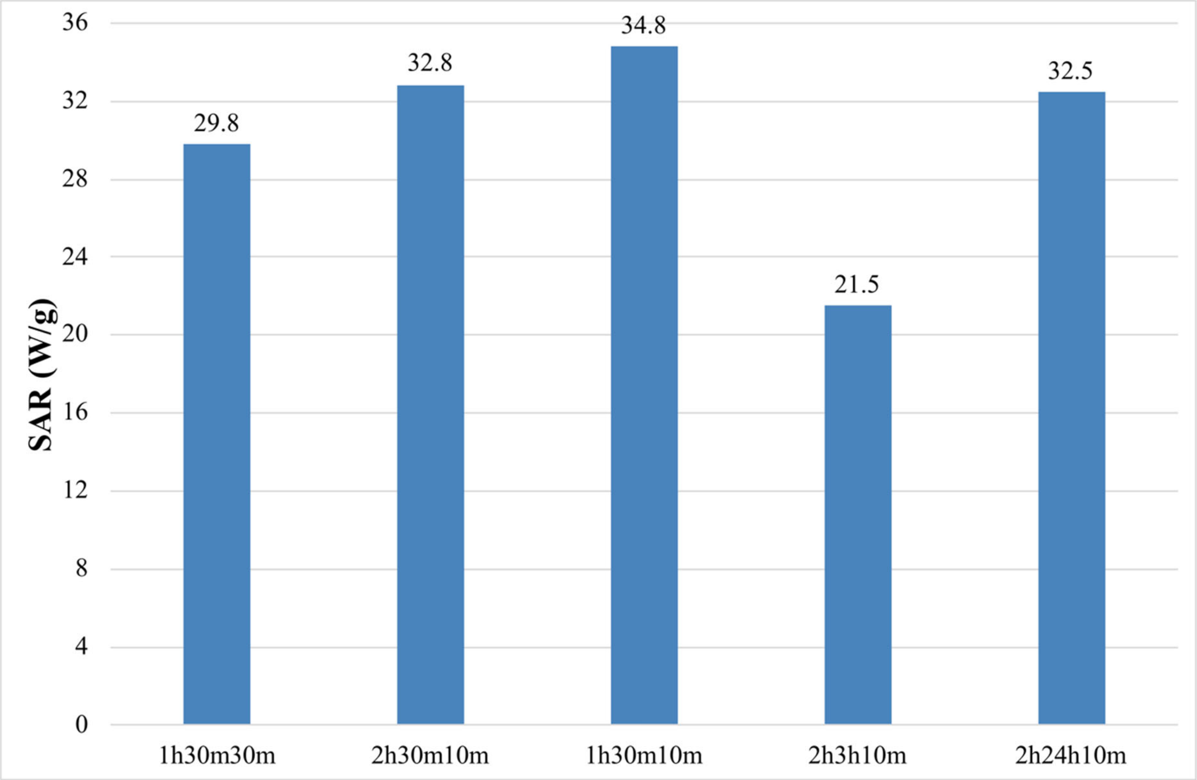

Figure S7: SAR values of samples at 109.4 kHz and 20.56 mT.

Electron spin resonance

Commercial suspension (m=6.1mg) was placed in the ESR spectrometer, whose iron oxide mass content is $m_{\text{Comm}} = 6.1\text{mg} \cdot \frac{c}{\rho} = 1.16\text{ m}$. The investigated lyophilized sample had a mass of 16.5 mg, which was placed in an ESR quartz tube as a powder. Fig. 1. shows the derivate ESR spectra of the reference and the investigated lyophilized sample. In general, the mass ratio of the iron oxide in the two samples can be obtained by double integration of the curves but as the overall shape of the curves match each other well, the relative iron oxide mass ratio is simply the ratio of the peak-to-peak signals. This is $\frac{\text{Lyophilized signal peak−to−peak}}{\text{Reference signal peak-to-peak}} = 0.68$, which means that the iron oxide content of the investigated lyophilized sample is $m_{\text{Lyo}} = 0.68\, m_{\text{Comm}} = 0.79$ mg. This yields a iron oxide mass concentration in the lyophilized sample of 4.8 %.
